# High awareness, inadequate practices: a cross-sectional KAP study on Chagas disease in an endemic Venezuelan community

**DOI:** 10.1186/s41182-025-00868-5

**Published:** 2025-12-12

**Authors:** Iván A. Escalante-Pérez, Fhabián S. Carrión-Nessi, Grecia de J. Erimee-Vieira, Mariana de J. de Marchis-Vento, Óscar D. Omaña-Ávila, Mario A. Dubuc-Ponte, Daniela I. Castro-Betancourt, Vanessa C. Sande-Mujica, Juan M. Contreras-Rengifo, Rachell A. Molina-Mendoza, Alejandro M. Loreto-Rodrigues, Juan C. Gomes-González, René Haddad-Soto, Daniela L. Mendoza-Millán, Belkisyolé Alarcón de Noya, David A. Forero-Peña

**Affiliations:** 1 Biomedical Research and Therapeutic Vaccines Institute, Ciudad Bolívar, Venezuela; 2 School of Medicine, Universidad Central de Venezuela, Caracas, Venezuela; 3 “Vive Más” Foundation, Caracas, Venezuela; 4 Navarra Center for International Development, Institute for Culture and Society, Universidad de Navarra, Pamplona, Spain; 5 Immunology Section, Instituto de Medicina Tropical “Dr. Félix Pifano”, Universidad Central de Venezuela, Caracas, Venezuela; 6 Department of Infectious Diseases, Hospital Universitario de Caracas, Caracas, Venezuela

**Keywords:** Chagas disease, *Trypanosoma cruzi*, Health knowledge, Attitudes, Practice, Cross-sectional studies, Venezuela, Rural population, Epidemiology

## Abstract

**Background:**

Chagas disease, caused by *Trypanosoma cruzi*, remains a significant public health problem in Venezuela, with evidence of re-emerging transmission and increasing concern over non-vectorial routes such as oral and congenital transmission. Effective public health strategies require a clear understanding of community-level knowledge, attitudes, and practices (KAP). This study aimed to characterize and identify predictors of KAP regarding Chagas disease in a rural, endemic community in Portuguesa state, Venezuela.

**Methods:**

A cross-sectional survey was conducted in September 2024 in the “Virgen de Coromoto” community. Participants aged 18 and older were recruited via non-probabilistic sampling. Data were collected using a pre-validated 57-item questionnaire covering sociodemographic aspects, knowledge, attitudes, and practices. KAP levels were categorized as high/low, positive/negative, and appropriate/inappropriate using a data-driven two-step cluster analysis. Binomial logistic regression was used to identify factors associated with these KAP outcomes.

**Results:**

A total of 317 individuals participated in the study. The median age was 36 years, and 57.7% (*n* = 183) were female. While a majority of participants demonstrated high knowledge (59.3%) and positive attitudes (57.4%), a significant proportion engaged in inappropriate preventive practices (61.8%). Knowledge deficits were identified concerning non-vectorial transmission routes, with only 24.9% correctly identifying contaminated food/juices and 40.7% identifying blood transfusions as risks. Women of reproductive age had significantly lower knowledge scores compared to the rest of the population (median score 9 vs. 11, *p* < 0.001). Being a woman of reproductive age (aOR = 1.75, 95% CI = 1.04–2.95, *p* = 0.034) and having negative attitudes (aOR = 1.82, 95% CI = 1.09–3.03, *p* = 0.021) were significant predictors of low knowledge. Having metallic screens on windows and doors was associated with a lower likelihood of inappropriate practices (aOR = 0.46, 95% CI = 0.22–0.97, *p* = 0.04).

**Conclusions:**

Despite generally high awareness, a critical disconnect exists between knowledge and protective behaviours in this endemic community. Specific vulnerabilities, including poor understanding of oral and congenital transmission routes and lower knowledge among women of reproductive age, pose significant risks. These findings underscore the need for targeted, evidence-based educational interventions that move beyond general awareness to address specific behavioural barriers and protect vulnerable groups.

**Supplementary Information:**

The online version contains supplementary material available at 10.1186/s41182-025-00868-5.

## Background

Chagas disease (CD), a parasitic infection caused by *Trypanosoma cruzi*, is a major neglected tropical disease affecting more than 7 million people worldwide, primarily in Latin America [1]. The disease is intrinsically linked to socioeconomic vulnerability, with transmission historically associated with poor housing conditions in rural areas that facilitate contact with triatomine vectors [2]. In recent decades, the epidemiological landscape has shifted due to migration, urbanization, and ecological changes [3–5], leading to the emergence of CD as a global health issue and an increased importance of non-vectorial transmission routes, including congenital, transfusional, and oral transmission [6–9].

In Venezuela, CD represents a persistent and evolving public health problem. After decades of successful control programs, evidence points to a re-emergence of active transmission in rural and urban areas [10–12]. This situation is compounded by the increasing frequency of acute CD outbreaks linked to oral transmission through contaminated food and juices [2, 13], a phenomenon that alters traditional risk perceptions and requires different prevention strategies than vector control alone [3]. These developments underscore the urgent need to understand how at-risk communities perceive, understand, and act upon the complex risks associated with CD today.

Knowledge, attitudes, and practices (KAP) studies are crucial tools for identifying gaps in public understanding and barriers to effective prevention [14]. Previous research in Latin America, including studies in the Portuguesa state of Venezuela, has consistently identified deficiencies in community knowledge and suboptimal preventive practices [15–17]. However, critical questions remain. The reasons for the frequent disconnect between positive community attitudes towards prevention and the persistence of high-risk behaviours are not well understood. Furthermore, there is a specific lack of detailed characterisation of the knowledge held by women of reproductive age (WRA), a group of paramount importance for the prevention of congenital transmission [18], which is a key pathway for the perpetuation of CD in both endemic and non-endemic regions [19].

Therefore, this study aimed to build upon previous findings by providing a deeper characterization of the KAP related to CD in a rural community in Portuguesa, Venezuela. The specific objectives were: (1) to assess the current levels of KAP using a validated instrument; (2) to identify sociodemographic and experiential factors associated with knowledge gaps and inappropriate practices; and (3) to perform a focused analysis of the knowledge levels among WRA to better inform strategies aimed at interrupting congenital transmission.

## Methods

### Study site

**Fig. 1:**
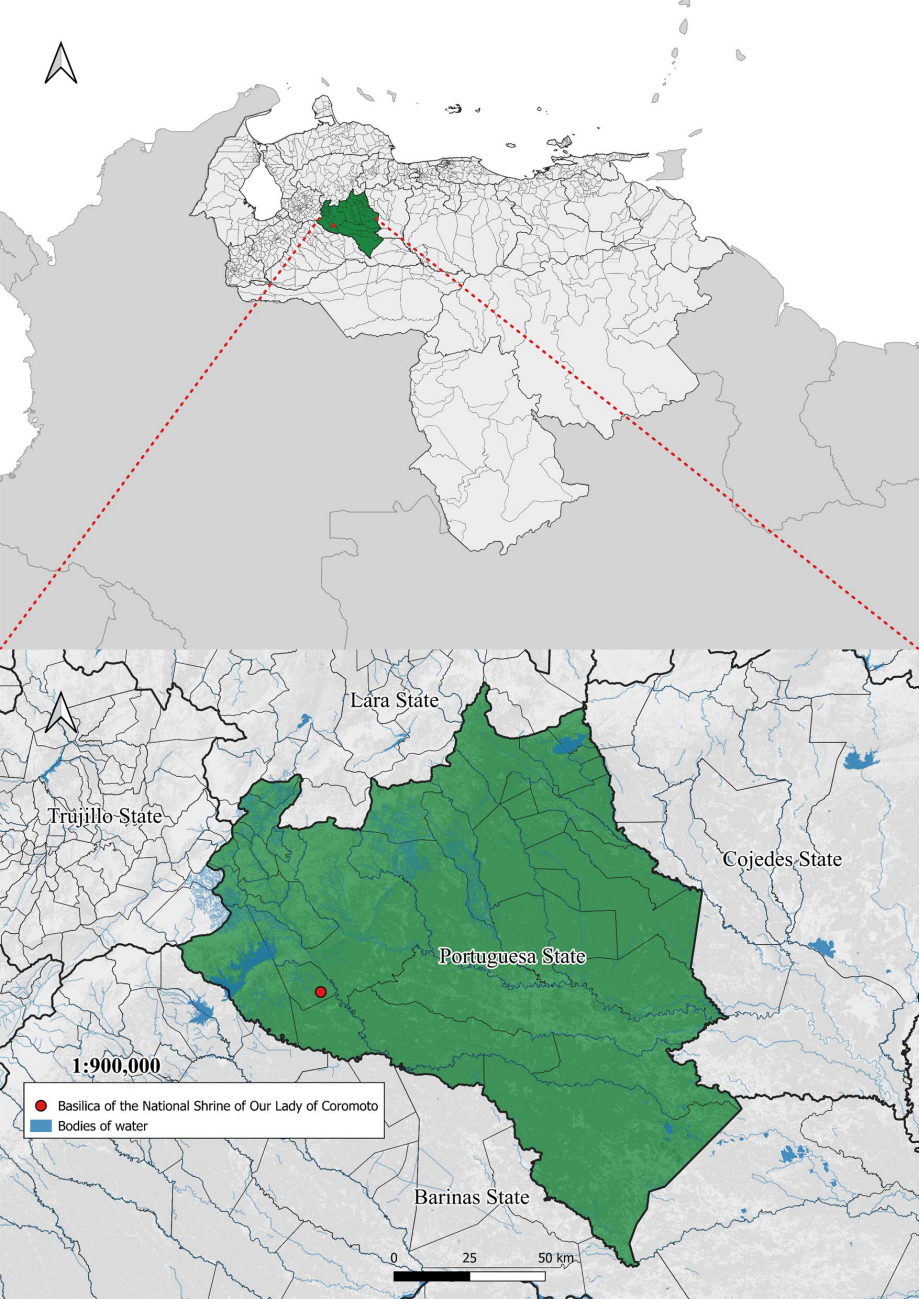
Location of Portuguesa state and the Basilica of the National Shrine of Our Lady of Coromoto, Guanare parish, Guanare municipality, Portuguesa state, Venezuela

This study was conducted in “Virgen de Coromoto” community, a rural settlement of 5,725 inhabitants located 11 km east of Guanare parish [20], part of Guanare municipality located in the northwestern part of Portuguesa state in Venezuela’s central-western region (08°52′36′′ to 09°26′44′′ N, 69°25′55′′ to 69°58′50′′ W), characterized by a tropical climate with temperatures ranging from 24 to 28°C, at a height of 183 MASL (Fig. 1). To the best of our knowledge, the 1990 national census, the last one to focus on housing materials, revealed that approximately ¼ of the 22,803 houses in the municipality were classified as farmhouses or rural dwellings. Among these, more than ¾ had outer walls constructed of adobe and wattle without additional framing, 60% had dirt floors and 689 had roofs made of palms or reeds [21].

### Study design and participant recruitment

A cross-sectional study was conducted from September 3 to 7, 2024, at the “Virgen de Coromoto” community during a medical mission that took place in the facilities of the Basilica of the National Shrine of Our Lady of Coromoto (Fig. 1). One month prior to data collection, the research team, comprised of volunteer physicians from the “Vive Más” Foundation, engaged with community leaders and local healthcare workers (HCWs) to explain the study’s objectives and encourage participation through biweekly meetings with household heads and weekly routine home visits by local HCWs. All individuals aged 18 years or older who attended the medical mission were invited to participate. A total of 508 participants were registered, 330 of whom were adults (81.51% female). After explaining the methodology and approach of the study, 96.1% expressed their desire to be included and signed informed consent form. A non-probabilistic convenience sampling method was employed. For subsequent analysis, participants were categorized into two groups: WRA (18–44 years) and the remaining population (men of all ages and women > 44 years).

### Survey instrument development and validation

A structured KAP questionnaire was developed based on previous studies [22–24] and guidelines from the PAHO/WHO [25] and the CDC [26]. The instrument was developed in Spanish and validated by a panel of experts in epidemiology, infectious diseases, parasitology, and research methodology.

An initial pilot test with 231 participants in July 2024 revealed low internal consistency for the KAP sections (Cronbach’s alpha = 0.37, 0.36, and 0.36, respectively). Following revisions to item content and wording by the expert panel, a second pilot test was administered online to 78 community members. This revised instrument demonstrated significantly improved reliability for the knowledge section (Cronbach’s alpha = 0.80, Kuder-Richardson 20 = 0.97) and the attitudes section (Cronbach’s alpha = 0.80), with a minor improvement for the practices section (Cronbach’s alpha = 0.39). The lower internal consistency for the practices scale is likely attributable to the multidimensional nature of the preventive behaviours assessed.

The final 57-item questionnaire comprised four sections:

1. Sociodemographics (23 items): age, sex, education, occupation, household composition, and housing materials.
2. Knowledge (18 items): multiple-choice questions (yes/no/don’t know) on the vector, transmission routes, symptoms, treatment, and prevention.
3. Attitudes (6 items): questions assessed on a 5-point Likert scale from “completely agree” to “completely disagree”.
4. Practices (7 items): questions assessed on a 5-point frequency scale from “always” to “never”.

To ensure accurate vector identification, participants were shown photographs of the primary local triatomine species (*Rhodnius prolixus*, *Panstrongylus geniculatus*, and *Triatoma maculata*).

### Data collection procedures

Data was collected through structured, face-to-face interviews conducted by trained members of the research team. To ensure standardisation and to minimise bias, all interviewers completed a half-day training workshop covering the study protocol, ethical conduct, and standardised administration of the questionnaire. A standardised script was used to introduce the study, obtain informed consent, and ask each question verbatim. Interviews were conducted in private, quiet rooms to ensure confidentiality. Participants were assured of the anonymity of their responses to mitigate social desirability bias. The average interview duration was 20 min.

### Data analysis

To avoid arbitrary cut-offs (e.g., median split) for categorizing KAP scores, a two-step cluster analysis was employed to identify naturally occurring groups within the data. This statistical technique is used in public health research to segment populations into homogeneous groups based on a set of variables [27]. Using a log-likelihood distance measure, the algorithm first pre-clustered cases and then used a hierarchical clustering method to determine the optimal number of clusters based on Schwarz’s Bayesian information criterion. This data-driven approach produced two distinct clusters for each KAP domain, which were profiled by their mean scores and designated as “low” vs. “high” knowledge, “negative” vs. “positive” attitudes, and “inappropriate” vs. “appropriate” practices.

Data were summarised using descriptive statistics (mean, standard deviation, median, interquartile range, frequencies, and percentages). The Kolmogorov–Smirnov test was used to assess the normality of numerical variables. The Mann–Whitney U test (for non-normal data) and Student’s *t*-test (for normal data) were used for group comparisons. Categorical variables were analysed using the chi-square test or Fisher’s exact test. A *p*-value of < 0.05 was considered statistically significant, with Bonferroni correction applied for *post-hoc* analyses. To identify predictors of KAP outcomes, binomial logistic regression models were constructed. Model fit was assessed using Nagelkerke’s R^2^ and the Hosmer–Lemeshow test. All analyses were performed using SPSS version 27, GraphPad Prism version 10.1.2, and R version 4.5.0.

## Results

### Participant sociodemographics

A total of 317 individuals participated in the study. The median age was 36 years, and 57.7% (*n* = 183) were female. Out of the female participants, 122 were of reproductive age (18–44 years), hereafter referred to as the “WRA” group. The remaining 195 participants (men and women > 44 years) are referred to as the “rest of the population” group. Key sociodemographic characteristics are detailed in Table 1. The WRA group was significantly younger than the rest of the population group (median age 30 vs. 46 years, *p* < 0.001). A significantly higher proportion of the WRA group had completed secondary education (51.6% vs. 35.9% in the rest of the population group, *p* < 0.001), while a higher proportion of the rest of the population group had no formal education (2.5% vs. 14.4% in the WRA group, *p* < 0.001).

**Table 1:** Sociodemographic characteristics of participants

Characteristics	Total (*n* = 317, 100%)	WRA (*n* = 122, 38.5%)	Rest (*n* = 195, 61.5%)	*p*-value
Age, median (IQR), years	36 (26–50)	30 (23–36)	46 (32–53)	< 0.001^*^
Sex, *n* (%)				
Male	134 (68.7)	0 (0)	134 (68.7)	**< 0.001**^**†**^
Female	183 (57.7)	122 (100)	61 (31.3)	
Education level, *n* (%)				
None	31 (9.8)	3 (2.5)	28 (14.4)	**< 0.001**^**†§**^
Primary	54 (17)	15 (12.3)	39 (20)	
Secondary	133 (42)	63 (51.6)	70 (35.9)	
University	99 (31.2)	41 (33.6)	58 (29.7)	
Occupation, *n* (%)				
Services and trades	90 (28.4)	30 (24.6)	60 (30.8)	**< 0.001**^**†||**^
Housekeeper	60 (18.9)	32 (26.2)	28 (14.4)	
Public servant	50 (15.8)	14 (11.5)	36 (18.5)	
Merchant	39 (12.3)	17 (13.9)	22 (11.3)	
Teacher	38 (12.3)	18 (14.8)	20 (10.3)	
Farmer	22 (6.9)	0 (0)	22 (11.3)	
Student	18 (5.7)	11 (9)	7 (3.6)	
Do you have metallic screens on the doors and windows of your home?, yes (%)	61 (19.2)	18 (14.8)	43 (22.1)	0.109^†^
Have you seen the insect that transmits this disease?, yes (%)	160 (50.5)	47 (38.5)	113 (57.9)	**< 0.001**^**†**^
Inside the house: bedroom	47 (29.4)	15 (31.9)	32 (28.3)	0.649^†^
Inside the house: kitchen	18 (11.3)	5 (10.6)	13 (11.5)	0.875^†^
Inside the house: bathroom	9 (5.6)	1 (2.1)	8 (7.1)	0.216^†^
Peridomiciliary	90 (56.3)	31 (66)	59 (52.2)	0.11^†^
Where the dog/cat rests	1 (0.6)	0 (0)	1 (0.9)	0.706^‡^
In the chicken coop	6 (3.8)	2 (4.3)	4 (3.5)	0.57^‡^
In a park	33 (20.6)	6 (12.8)	27 (23.9)	0.113^†^
Other	39 (24.4)	7 (14.9)	32 (28.3)	0.072^†^
Have you been bitten by this insect?, yes (%)	9 (2.8)	2 (1.6)	7 (3.6)	0.309^†^
Did you go to a health centre?, yes (%)	5 (55.6)	1 (50)	4 (57.1)	0.722^‡^
Were you diagnosed with CD at that centre?, yes (%)	1 (20)	0 (0)	1 (25)	0.8^‡^

^*^Mann–Whitney U test, ^†^chi-square test, ^‡^Fisher’s exact test, ^§^Significant association only between None and Rest (*p* = 0.00052) and between Secondary and WRA (*p* = 0.00572) for a value of α = 0.00625 by Bonferroni correction, ^||^Significant association only between Farmer and Rest (*p* = 0.00012) for a value of α = 0.00357 by Bonferroni correction. *WRA* women of reproductive age, *IQR* interquartile range, *CD* Chagas disease

Regarding housing and environmental factors, 50.5% of participants reported having seen the vector, with sightings being significantly more common among the rest of the population group (57.9% vs. 38.5% in the WRA group, *p* < 0.001). The most common sighting location was the peridomiciliary area (56.3%) (Table 1). Only 19.2% of households had metallic screens on doors and windows. Most participants lived in homes with block walls (87.7%), zinc roofs (75.4%), and cement floors (75.1%). The presence of domestic animals was high, with 77.6% reporting dogs and 53.3% reporting cats. Consumption of sugarcane juice was significantly more prevalent in the rest of the population group (46.2% vs. 27% in the WRA group, *p* < 0.001). Regarding sources of information, only 14.5% of participants reported having learned about the disease from community HCWs (Supplementary Data 1).

### Knowledge of Chagas disease

Based on the cluster analysis, 59.3% (*n* = 188) of participants were categorized as having “high” knowledge (score ≥ 10), while 40.7% (*n* = 129) had “low” knowledge. The median knowledge score for the entire sample was 10. The WRA group had a significantly lower median knowledge score (9) compared to the rest of the population group (11, *p* < 0.001). Consequently, a higher proportion of the WRA group was classified as having low knowledge (50.8% vs. 34.4% in the rest of the population group, *p* = 0.004). Specific knowledge items are detailed in Table 2. While general knowledge about the vector (86.8% correct) and cardiac symptoms (80.1% correct) was high, significant deficits were observed for non-vectorial transmission routes. Only 24.9% correctly identified contaminated juices as a transmission route, and only 40.7% correctly identified blood transfusions. Knowledge of congenital transmission was also low (36.6% correct). The WRA group demonstrated significantly lower knowledge than the rest of the population group regarding transmission via contaminated juices (*p* = 0.002) and blood transfusions (*p* = 0.001), the development of cardiac complications (*p* = 0.025), and the existence of an asymptomatic phase (*p* = 0.001).

**Table 2:** Knowledge about Chagas disease

Knowledge	Total (*n* = 317, 100%)	WRA (*n* = 122, 38.5%)	Rest (*n* = 195, 61.5%)	*p*-value
Knowledge, median (IQR), points	10 (8–11)	9 (7–11)	11 (9–12)	< 0.001^*^
Knowledge, *n* (%)				
Low (≤ 9 points)	129 (40.7)	62 (50.8)	67 (34.4)	**0.004**^**†**^
High (≥ 10 points)	188 (59.3)	60 (49.2)	128 (65.6)	
K1. How is CD transmitted?, correct (%)				
K1.1. By the bite of a mosquito	141 (44.5)	58 (47.5)	83 (42.6)	0.386^†^
K1.2. Through contaminated food	88 (27.8)	29 (23.8)	59 (30.3)	0.21^†^
K1.3. Through blood transfusions	129 (40.7)	36 (29.5)	93 (47.7)	**0.001**^**†**^
K1.4. Through physical contact with infected people	190 (59.9)	68 (55.7)	122 (62.6)	0.228^†^
K1.5. From mother to child during pregnancy	116 (36.6)	38 (31.1)	78 (40)	0.111^†^
K2. Is CD caused by a virus?, correct (%)	175 (55.2)	67 (54.9)	108 (55.4)	0.935^†^
K3. Can CD be transmitted by consuming contaminated juices?, correct (%)	79 (24.9)	19 (15.6)	60 (30.8)	**0.002**^**†**^
K4. Is CD transmitted by bed bugs?, correct (%)	112 (35.3)	32 (26.2)	80 (41)	**0.007**^**†**^
K5. Can CD cause enlargement of the heart?, correct (%)	254 (80.1)	90 (73.8)	164 (84.1)	**0.025**^**†**^
K6. Is the insect that transmits CD in Venezuela known as “kissing bug”?, correct (%)	275 (86.8)	101 (82.8)	174 (89.2)	0.1^†^
K7. Do palm roofs, bahareque walls, and cracks in the walls favour the presence of the insect that transmits CD?, correct (%)	270 (85.2)	97 (79.5)	173 (88.7)	**0.025**^**†**^
K8. Is cleaning and maintaining house hygiene a way to prevent CD?, correct (%)	295 (93.1)	114 (93.4)	181 (92.8)	0.832^†^
K9. Is fumigation a form of prevention for CD?, correct (%)	288 (90.9)	107 (87.7)	181 (92.8)	0.124^†^
K10. Does having animals like opossums, dogs, cats, or chickens near the house increase the probability of contracting CD?, correct (%)	170 (53.6)	64 (52.5)	106 (54.4)	0.741^†^
K11. Is there a treatment for CD?, correct (%)	191 (60.3)	69 (56.6)	122 (62.6)	0.288^†^
K12. Is there a stage of CD where people may not have symptoms?, correct (%)	172 (54.3)	52 (42.6)	120 (61.5)	**0.001**^**†**^
K13. Can CD be diagnosed with a patient’s stool sample?, correct (%)	91 (28.7)	32 (26.2)	59 (30.3)	0.441^†^

^*^Mann–Whitney U test, ^†^chi-square test. *WRA* women of reproductive age, *IQR* interquartile range, *CD* Chagas disease

### Attitudes towards Chagas disease

**Fig. 2:**
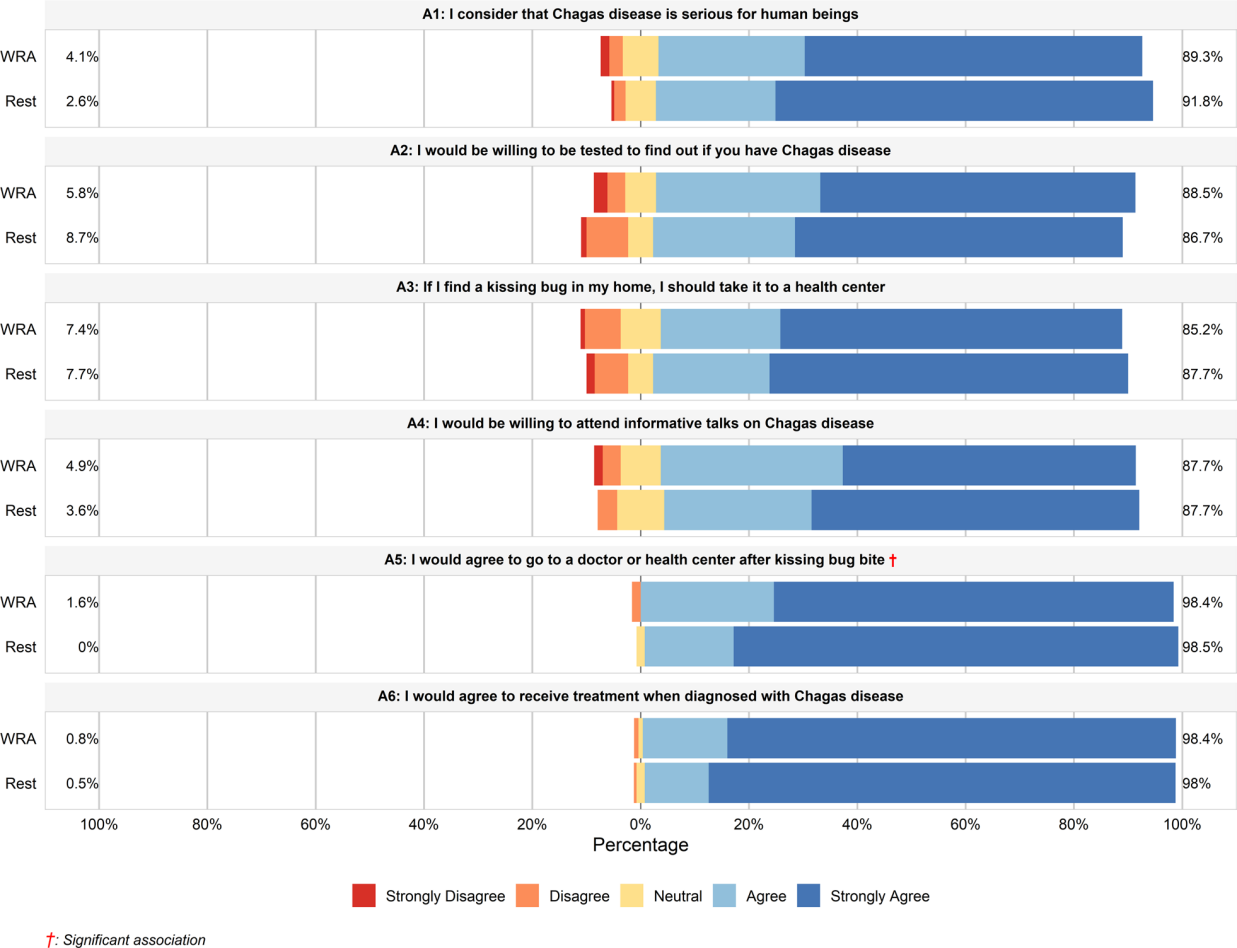
Attitudes towards Chagas disease among the WRA group and the rest of the population group. The bars indicate the proportion of responses for each statement, stratified by participant group (WRA and rest of the population). Positive attitudes (agree/strongly agree) are represented towards the right in shades of blue, while negative attitudes (disagree/strongly disagree) are represented towards the left in shades of red and orange

Overall, 57.4% (*n* = 182) of participants were categorized as having “positive” attitudes (score ≥ 28). There was no significant difference in median attitude scores or the proportion of positive/negative attitudes between the WRA group and the rest of the population group. As shown in Fig. 2 and Supplementary Data 2, attitudes were generally positive across specific items. A large majority of participants agreed or strongly agreed that CD is a serious illness (90.9%), that they would be willing to be tested (87.4%), and that they would seek medical attention after a vector bite (98.5%). Similarly, 98.1% would be willing to receive treatment if diagnosed.

### Practices regarding Chagas disease prevention

**Fig. 3:**
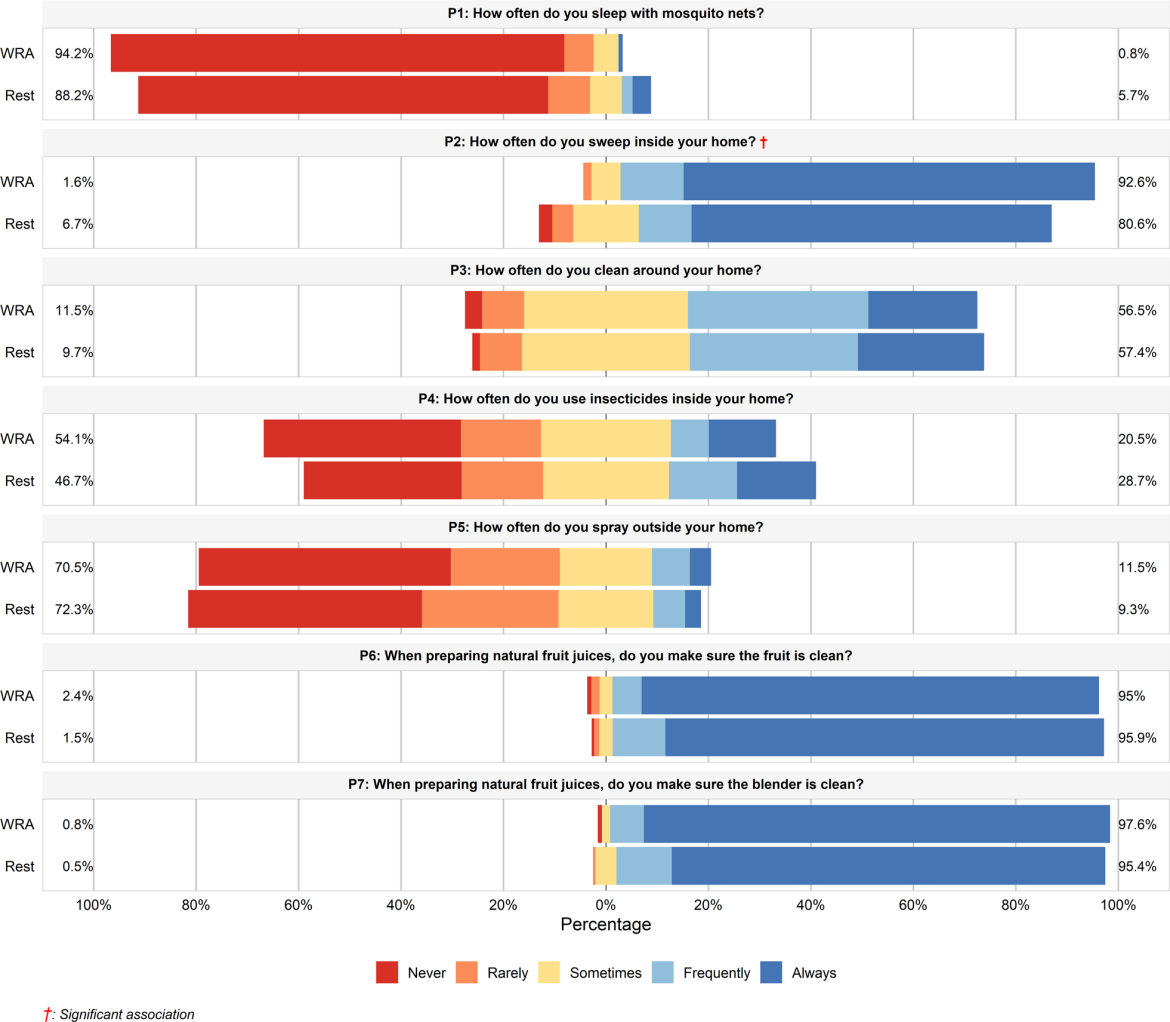
Practices towards Chagas disease among the WRA group and the rest of the population group. The bars indicate the proportion of responses for each statement, stratified by participant group (WRA and rest of the population). Appropriate practices (agree/strongly agree) are represented towards the right in shades of blue, while inappropriate practices (disagree/strongly disagree) are represented towards the left in shades of red and orange

A majority of participants (61.8%, *n* = 196) were categorized as having “inappropriate” practices (score ≤ 24). There was no significant difference in median practice scores or the proportion of appropriate/inappropriate practices between the two study groups. Specific preventive practices are detailed in Fig. 3 and Supplementary Data 3. Adherence to key vector control measures was low. A vast majority of participants (83.3%) reported “never” sleeping under a mosquito net. Similarly, 47% reported “never” fumigating outside their homes, and 33.8% reported “never” using insecticides inside. Most participants reported “always” cleaning inside their homes (74.1%). Practices related to food hygiene were high, with 87.1% reporting they “always” wash fruits before preparing juices.

### Factors associated with KAP outcomes

Results from the binomial logistic regression models are presented in Table 3.

- Low knowledge: factors associated with a higher likelihood of having low knowledge were being in the WRA group (aOR = 1.75, 95% CI = 1.04–2.95, *p* = 0.034), having negative attitudes (aOR = 1.82, 95% CI = 1.09–3.03, *p* = 0.021), and self-perceiving one’s knowledge as “very bad” (aOR = 2.83, 95% CI = 1.44–5.59, *p* = 0.003).
- Negative attitudes: factors associated with a lower likelihood of having negative attitudes included having seen the vector in the bedroom (aOR = 0.24, 95% CI = 0.10–0.58, *p* = 0.001), having a family member with CD (aOR = 0.24, 95% CI = 0.10–0.58, *p* = 0.002), and receiving information from radio/TV (aOR = 0.17, 95% CI = 0.05–0.54, *p* = 0.003) or social media (aOR = 0.24, 95% CI = 0.10–0.58, *p* = 0.003). Living in a household with ≥ 6 people was associated with a higher likelihood of negative attitudes (aOR = 2.82, 95% CI = 1.11–7.13, *p* = 0.029).
- Inappropriate practices: factors associated with a lower likelihood of having inappropriate practices were having metallic screens on doors and windows (OR = 0.46, 95% CI 0.22–0.97, *p* = 0.04) and having seen the vector in a location other than the bedroom (aOR = 0.43, 95% CI = 0.20–0.91, *p* = 0.026).

**Table 3:** Factors associated with knowledge, attitudes, and practices regarding Chagas disease

Factors	β	*p*-value	aOR (95% CI)
Low knowledge^*^			
How do you consider your knowledge about CD?: very poor	1.042	**0.003**	2.834 (1.437–5.591)
Negative attitudes	0.599	**0.021**	1.821 (1.093–3.034)
WRA	0.562	**0.034**	1.754 (1.043–2.951)
Negative attitudes^†^			
Where did you acquire your knowledge about CD?: radio and/or television	−1.776	**0.003**	0.169 (0.053–0.539)
Where did you acquire your knowledge about CD?: social media	−1.433	**0.003**	0.239 (0.098–0.579)
How many people live in your house?: ≥ 6 people	1.035	**0.029**	2.816 (1.112–7.132)
Have you seen the insect that transmits this disease?: inside the house: bedroom	−1.413	**0.001**	0.243 (0.102–0.582)
Do you have relatives with CD?: yes	−1.433	**0.002**	0.239 (0.098–0.579)
Inappropriate practices^‡^			
Do you have metal screens on the doors and windows of your home?: yes	−0.774	**0.04**	0.461 (0.221–0.965)
Have you seen the insect that transmits this disease?: other	−0.844	**0.026**	0.43 (0.204–0.905)

^*^p < 0.001, Nagelkerke’s R^2^ = 0.24, Hosmer–Lemeshow test = 0.573; ^†^*p* < 0.001, Nagelkerke’s R^2^ = 0.307, Hosmer–Lemeshow test = 0.415; ^‡^*p* = 0.009, Nagelkerke’s R^2^ = 0.078, Hosmer–Lemeshow test = 0.954. *aOR* adjusted odds ratio, *CD* Chagas disease, *WRA* women of reproductive age

## Discussion

This study provides a critical snapshot of the KAP regarding CD in a rural, endemic Venezuelan community, revealing a significant “KAP gap”. The observed disconnect between high knowledge and inadequate practice is a persistent and defining challenge for CD control across Latin America [15, 16, 23]. Our findings starkly illustrate this paradox: despite a majority having high knowledge (59.3%) and positive attitudes, over 83% of participants never use bed nets and nearly half never fumigate outdoors. This paradox is particularly troubling when contextualised with other regional studies. For instance, while a study in the Colombian Caribbean found poor practices were linked to extremely low knowledge levels (86% of participants having “very poor” knowledge) [16], our community exhibited poor practices despite high general awareness. This suggests that simply elevating general awareness is an insufficient strategy to drive behavioural change. The behavioural inertia appears to be a complex phenomenon rooted in the interplay of three key factors: the nature of the community’s knowledge, their perception of risk, and prevailing socioeconomic realities [16, 28]. First, the “high knowledge” observed may be superficial, representing general awareness of the vector without a deep,, internalised understanding of the disease’s life-threatening potential; communities may recognise the vector but consider it a mere nuisance rather than a serious health threat [29]. Second, in a hyperendemic region, long-term exposure may lead to a normalisation of risk or fatalism, where the decades-long latency of chronic symptoms diminishes the perceived urgency of daily prevention [30, 31]. Finally, these cognitive and perceptual factors are compounded by tangible socioeconomic barriers. For resource-strained households, the immediate costs of prevention—such as purchasing insecticides or enduring the discomfort of bed nets in a tropical climate—may outweigh the perceived benefits of mitigating a distant health threat [32]. This calculus, while detrimental to long-term health, is a rational response to competing daily priorities. Our finding that living in a larger household (≥ 6 people) was associated with more negative attitudes may further reflect this resource strain and overcrowding. Therefore, effective educational programs must move beyond general awareness to address specific, high-risk gaps, such as the asymptomatic nature of the chronic phase and non-vectorial transmission routes [6, 33, 34].

Perhaps the most alarming finding is the profound ignorance surrounding oral transmission, a critical failure of public health communication that leaves the community dangerously exposed. Only a quarter of participants (24.9%) identified contaminated food or juices as a transmission route. This knowledge deficit is particularly grave in Venezuela, a country that has documented at least 10 distinct oral CD outbreaks since 2007, several of which were lethal [2]. Crucially, many of these outbreaks have been explicitly linked to the consumption of contaminated, unpasteurized fruit juices—including guava, passion fruit, and mango juice—which are widely consumed by our study population (69.7% report consuming such juices) [2]. The risk is not merely in the fruit itself but in the preparation process. Outbreak investigations have shown that contamination often occurs when juices are prepared in large batches and the boiled fruits are left to cool in open containers, typically overnight, allowing vectors attracted to light to fall into the containers and be macerated during blending [2]. Therefore, the community’s reported high adherence to washing fruit (87.1%), while a positive general hygiene practice, provides a false sense of security and is insufficient to mitigate the primary risk of oral transmission. This specific gap between the actual transmission mechanism and the community’s preventive measures represents a major, unaddressed vulnerability that requires urgent, highly specific educational intervention focused on safe food and beverage handling practices.

Equally concerning are the specific vulnerabilities identified among WRA. Our finding that WRA have significantly lower overall knowledge scores than the rest of the population is a perilous trend, as this group is central to the generational persistence of CD. This vulnerability is most evident in the low awareness of congenital transmission (36.6% correct). While poor knowledge of this route is a documented problem elsewhere [35], our study reveals a more alarming dynamic: the very group responsible for interrupting this transmission cycle is the least informed within their own community. This knowledge deficit represents a fundamental, grassroots barrier to achieving the goals of major international health initiatives, such as the PAHO/WHO “EMTCT-Plus” framework, which aims to eliminate mother-to-child transmission of CD alongside HIV, syphilis, and hepatitis B [36]. The framework’s strategy relies on integrating screening into routine antenatal care, but its success is predicated on awareness and acceptance within the target population. Our findings demonstrate that this foundational awareness is dangerously weak. This lack of community-level knowledge is the upstream cause of the downstream failures seen in clinical settings, where studies have documented alarmingly low adherence to screening protocols, poor newborn follow-up, and high rates of loss-to-follow-up for postpartum maternal treatment [37]. Furthermore, this issue is distinctly gendered. In this community, women are situated at a nexus of risk: they are the biological route for congenital transmission and, as the primary food preparers, the last line of defence against oral transmission. That this same group is the least knowledgeable highlights a profound, gender-based vulnerability. Therefore, interventions cannot be generic; they must be gender-sensitive, empowering women with specific knowledge about the risks they uniquely manage and delivered through trusted channels such as maternal and child health services.

This study has several strengths, including the rigorous, multi-stage validation of the KAP questionnaire and the use of a data-driven two-step cluster analysis to define KAP levels, which avoids the arbitrary nature of median splits and enhances the objectivity of our categorisations [27]. However, the study’s limitations must be acknowledged. The use of a non-probabilistic, convenience sample from a single community limits the generalisability of our findings to other parts of Portuguesa state or Venezuela. Additionally, the cross-sectional design allows us to identify associations but not to establish causality. Methodologically, the low internal consistency of the practices scale (Cronbach’s alpha = 0.39) is a limitation. However, this statistical finding likely reflects the conceptual nature of “preventive practices” rather than a flaw in the instrument’s items. Unlike knowledge or attitudes, which may be treated as unified, reflective constructs, the set of preventive practices represents a formative index of disparate behaviours. There is no theoretical reason to expect that actions from different domains—such as personal protection (using a bed net), household hygiene (sweeping), and chemical control (fumigating)—should be highly correlated. A person may diligently perform one action while neglecting another for various practical or economic reasons. Therefore, while the composite “practices score” should be interpreted with caution, the individual items retain high face and content validity, as assessed by our expert panel, and proved essential for identifying the specific, actionable behavioural gaps that are the focus of our public health recommendations.

### Public health implications

The results of this study have direct implications for CD control policy in Venezuela.

1. Shift from general to targeted education: broad awareness campaigns are clearly insufficient. Interventions must be tailored to address the specific knowledge gaps and behavioural barriers identified. This includes developing educational materials on safe food and juice preparation and disseminating them through community channels.
2. Empower WRA: health education must be integrated into prenatal and family planning services, explicitly addressing congenital transmission, the importance of screening, and the availability of treatment for newborns.
3. Leverage HCWs: our finding that only 14.5% of participants received information from HCWs highlights a missed opportunity. Training and empowering local HCWs to be primary sources of accurate, up-to-date information on CD—including non-vectorial routes—is a critical and achievable strategy.
4. Adopt a One Health approach: the high prevalence of domestic and synanthropic animals (dogs, cats, opossums) reported by participants underscores the close interaction between human, animal, and environmental domains in the *T. cruzi* transmission cycle. Future surveillance and control strategies should adopt a “One Health” framework, integrating veterinary and environmental surveillance with human public health efforts [38]. The study was conducted at a religious centre, highlighting the potential for faith-based organizations to serve as trusted partners in disseminating health information and implementing community-based interventions [39].

### Future research

Future research should aim to explore the “why” behind the observed KAP gap. Qualitative studies employing in-depth interviews on focus groups could provide rich insights into the socioeconomic and cultural barriers that prevent knowledge from translating into practice. Studies designed to compare the level of knowledge among young men and women are needed to identify whether the knowledge gap is solely due to age (young people of both sexes have a low level of knowledge) or whether the gender gap persists within that age group. Furthermore, longitudinal intervention studies are needed to design and evaluate the effectiveness of the targeted educational programs proposed here.

## Conclusions

This study reveals a critical paradox in a community highly endemic for CD: while general awareness is high, preventive practices remain dangerously inadequate. This gap between knowledge and behaviour is exacerbated by a widespread lack of understanding of non-vectorial transmission routes, such as oral and congenital transmission, which represent a growing threat in Venezuela. The identification of WRA as a particularly vulnerable group due to lower knowledge levels highlights an urgent need for targeted intervention. The findings strongly suggest that current public health strategies are failing to address the modern realities of CD transmission. To effectively control the disease and prevent its re-emergence, Venezuela requires a paradigm shift towards evidence-based, targeted educational programs that empower communities with the specific knowledge and tools needed to protect themselves from this persistent and evolving threat.

## Supplementary Information

Supplementary material 1.

## Data Availability

The datasets used and/or analysed during the current study are available from the corresponding author on reasonable request.

## Abbreviations

CD: Chagas disease
KAP: Knowledge, attitudes, and practices
WRA: Women of reproductive age
HCWs: Healthcare workers
