# Supplementary material for: High awareness, inadequate practices: a cross-sectional KAP study on Chagas disease in an endemic Venezuelan community
Source: Trop Med Health. 2025 Dec 12;53:187. doi: 10.1186/s41182-025-00868-5 (PMC12707000; doi:10.1186/s41182-025-00868-5)
Supplement: Supplementary file 1 — Supplementary material 1. [file 41182_2025_868_MOESM1_ESM.docx]

**Supplementary Data 1.** Other sociodemographic characteristics of participants, self-perceived knowledge and sources of information

| **Characteristics** | **Total (*n* = 317, 100%)** | **WRA (*n* = 122, 38.5%)** | **Rest (*n* = 195, 61.5%)** | ***p*-value** |
| --- | --- | --- | --- | --- |
| How many people live in your house?, *n* (%) |  |  |  | 0.112^*^ |
| <5 people | 259 (81.7) | 105 (86.1) | 154 (79) |  |
| ≥6 people | 58 (18.3) | 17 (13.9) | 41 (21) |  |
| Dwelling |  |  |  |  |
| Wall, *n* (%) |  |  |  | 0.098^†^ |
| Block | 278 (87.7) | 104 (85.2) | 174 (89.2) |  |
| Mud | 13 (4.1) | 6 (4.9) | 7 (3.6) |  |
| Brick | 11 (3.5) | 2 (1.6) | 9 (4.6) |  |
| Zinc | 7 (2.2) | 4 (3.3) | 3 (1.5) |  |
| Bahareque | 6 (1.9) | 5 (4.1) | 1 (0.5) |  |
| Wood | 2 (0.6) | 1 (0.8) | 1 (0.5) |  |
| Roof, *n* (%) |  |  |  | 0.675^*^ |
| Zinc | 239 (75.4) | 95 (77.9) | 144 (73.8) |  |
| Concrete | 44 (13.9) | 16 (13.1) | 28 (14.4) |  |
| Tongue and groove wood | 34 (10.7) | 11 (9) | 23 (11.8) |  |
| Floor, *n* (%) |  |  |  | 0.788^†^ |
| Cement | 238 (75.1) | 94 (77) | 144 (73.8) |  |
| Tile | 69 (21.8) | 26 (21.3) | 43 (22.1) |  |
| Dirt | 9 (2.8) | 2 (1.6) | 7 (3.6) |  |
| Wood | 1 (0.3) | 0 (0) | 1 (0.5) |  |
| Outdoors (50 meters), yes (%) |  |  |  |  |
| Vegetation | 286 (90.2) | 112 (91.8) | 174 (89.2) | 0.453^*^ |
| Palm trees | 101 (31.9) | 38 (31.1) | 63 (32.3) | 0.829^*^ |
| Dogs | 246 (77.6) | 95 (77.9) | 151 (77.4) | 0.928^*^ |
| Cats | 169 (53.3) | 58 (47.5) | 111 (56.9) | 0.103^*^ |
| Chickens | 152 (47.9) | 57 (46.7) | 95 (48.7) | 0.729^*^ |
| Cows | 54 (17) | 21 (17.2) | 33 (16.9) | 0.947^*^ |
| Opossums | 136 (42.9) | 53 (43.4) | 83 (42.6) | 0.878^*^ |
| Do you consume natural juices (e.g., guava, passion fruit)?, yes (%) | 221 (69.7) | 92 (75.4) | 129 (66.2) | 0.081^*^ |
| Do you consume sugarcane juice?, yes (%) | 123 (38.8) | 33 (27) | 90 (46.2) | **<0.001^*^** |
| Do you have relatives with CD?, yes (%) | 72 (22.7) | 31 (25.4) | 41 (21) | 0.365^*^ |
| Degree of kinship (consanguinity), *n* (%) |  |  |  | 0.068^*^ |
| First | 21 (29.2) | 6 (19.4) | 15 (36.6) |  |
| Second | 26 (36.1) | 16 (51.6) | 10 (24.4) |  |
| Third | 19 (26.4) | 8 (25.8) | 11 (26.8) |  |
| Fourth | 6 (8.3) | 1 (3.2) | 5 (12.2) |  |
| State where the disease was acquired, *n* (%) |  |  |  | 0.806^†^ |
| Portuguesa | 62 (86.1) | 26 (83.9) | 36 (87.8) |  |
| Trujillo | 4 (5.6) | 3 (9.7) | 1 (2.4) |  |
| Barinas | 2 (2.8) | 1 (3.2) | 1 (2.4) |  |
| Distrito Capital | 2 (2.8) | 1 (3.2) | 1 (2.4) |  |
| Falcón | 1 (1.4) | 0 (0) | 1 (2.4) |  |
| Lara | 1 (1.4) | 0 (0) | 1 (2.4) |  |
| How do you rate your knowledge about CD?, *n* (%) |  |  |  | 0.434^*^ |
| Excellent | 3 (0.9) | 0 (0) | 3 (1.5) |  |
| Good | 17 (5.4) | 7 (5.7) | 10 (5.1) |  |
| Fair | 109 (34.4) | 39 (32) | 70 (35.9) |  |
| Poor | 108 (34.1) | 40 (32.8) | 68 (34.9) |  |
| Very poor | 80 (25.2) | 36 (29.5) | 44 (22.6) |  |
| Where did you get your knowledge about CD?, yes (%) |  |  |  |  |
| Family or close friends | 215 (67.8) | 78 (63.9) | 137 (70.3) | 0.241^*^ |
| During my academic training | 60 (18.9) | 22 (18) | 38 (19.5) | 0.748^*^ |
| Social media (TikTok, Facebook, Instagram) | 30 (9.5) | 12 (9.8) | 18 (9.2) | 0.858^*^ |
| On the internet (Google, health websites) | 32 (10.1) | 10 (8.2) | 22 (11.3) | 0.375^*^ |
| Radio and/or television | 48 (15.1) | 15 (12.3) | 33 (16.9) | 0.263^*^ |
| From community HCWs | 46 (14.5) | 18 (14.8) | 28 (14.4) | 0.923^*^ |
| None | 34 (10.7) | 18 (14.8) | 16 (8.2) | 0.067^*^ |
| Are there CD prevention and control services in your region?, *n* (%) |  |  |  | **0.02^*‡^** |
| Yes | 67 (21.1) | 16 (13.1) | 51 (26.2) |  |
| No | 112 (35.3) | 46 (37.7) | 66 (33.8) |  |
| Don’t know | 138 (43.5) | 60 (49.2) | 78 (40) |  |

^*^Chi-square test, ^†^Fisher’s exact test, ^‡^Significant association only between Yes and Rest (*p* = 0.00566) for a value of α = 0.00833 by Bonferroni correction. WRA: women of reproductive age, CD: Chagas disease, HCWs: healthcare workers

**Supplementary Data 2.** Attitudes towards Chagas disease

| **Attitudes** | **Total (*n* = 317, 100%)** | **WRA (*n* = 122, 38.5%)** | **Rest (*n* = 195, 61.5%)** | ***p*-value** |
| --- | --- | --- | --- | --- |
| Attitudes, median (IQR), points | 28 (26-30) | 28 (25-30) | 28 (26-30) | 0.315^*^ |
| Attitudes, *n* (%) |  |  |  |  |
| Negative (≤27 points) |  |  |  |  |
| Positive (≥28 points) |  |  |  |  |
| A1. Considers CD to be serious for humans, *n* (%) |  |  |  | 0.6^†^ |
| Strongly agree | 212 (66.9) | 76 (62.3) | 136 (69.7) |  |
| Agree | 76 (24) | 33 (27) | 43 (22.1) |  |
| Neutral | 19 (6) | 8 (6.6) | 11 (5.6) |  |
| Disagree | 7 (2.2) | 3 (2.5) | 4 (2.1) |  |
| Strongly disagree | 3 (0.9) | 2 (1.6) | 1 (0.5) |  |
| A2. Would be willing to get tested to know if you have CD, *n* (%) |  |  |  | 0.387^‡^ |
| Strongly agree | 189 (59.6) | 71 (58.2) | 118 (60.5) |  |
| Agree | 88 (27.8) | 37 (30.3) | 51 (26.2) |  |
| Neutral | 16 (5) | 7 (5.7) | 9 (4.6) |  |
| Disagree | 19 (6) | 4 (3.3) | 15 (7.7) |  |
| Strongly disagree | 5 (1.6) | 3 (2.5) | 2 (1) |  |
| A3. Believes that if a kissing bug is found in the home, it should be taken to a health centre, *n* (%) |  |  |  | 0.835^‡^ |
| Strongly agree | 206 (65) | 77 (63.1) | 129 (66.2) |  |
| Agree | 69 (21.8) | 27 (22.1) | 42 (21.5) |  |
| Neutral | 18 (5.7) | 9 (7.4) | 9 (4.6) |  |
| Disagree | 20 (6.3) | 8 (6.6) | 12 (6.2) |  |
| Strongly disagree | 4 (1.3) | 1 (0.8) | 3 (1.5) |  |
| A4. Would be willing to attend informational talks about CD, *n* (%) |  |  |  | 0.326^†^ |
| Strongly agree | 184 (58) | 66 (54.1) | 118 (60.5) |  |
| Agree | 94 (29.7) | 41 (33.6) | 53 (27.2) |  |
| Neutral | 26 (8.2) | 9 (7.4) | 17 (8.7) |  |
| Disagree | 11 (3.5) | 4 (3.3) | 7 (3.6) |  |
| Strongly disagree | 2 (0.6) | 2 (1.6) | 0 (0) |  |
| A5. Would agree to go to a doctor or health centre after a kissing bug bite, *n* (%) |  |  |  | 0.033^†^ |
| Strongly agree | 250 (78.9) | 90 (73.8) | 160 (82.1) |  |
| Agree | 62 (19.6) | 30 (24.6) | 32 (16.4) |  |
| Neutral | 3 (0.9) | 0 (0) | 3 (1.5) |  |
| Disagree | 2 (0.6) | 2 (1.6) | 0 (0) |  |
| Strongly disagree | 0 (0) | 0 (0) | 0 (0) |  |
| A6. Would agree to receive treatment if diagnosed with CD, *n* (%) |  |  |  | 0.681^†^ |
| Strongly agree | 269 (84.9) | 101 (82.8) | 168 (86.2) |  |
| Agree | 42 (13.2) | 19 (15.6) | 23 (11.8) |  |
| Neutral | 4 (1.3) | 1 (0.8) | 3 (1.5) |  |
| Disagree | 2 (0.6) | 1 (0.8) | 1 (0.5) |  |
| Strongly disagree | 0 (0) |  | 0 (0) |  |

^*^Mann-Whitney U test, ^†^Fisher’s exact test, ^‡^ chi-square test. WRA: women of reproductive age, IQR: interquartile range, CD: Chagas disease

**Supplementary Data 3.** Practices regarding Chagas disease prevention

| **Practices** | **Total (*n* = 317, 100%)** | **WRA (*n* = 122, 38.5%)** | **Rest (*n* = 195, 61.5%)** | ***p*-value** |
| --- | --- | --- | --- | --- |
| Practices, median (IQR), points | 24 (22-26) | 23 (22-26) | 24 (22-26) | 0.537^*^ |
| Practices, *n* (%) |  |  |  | 0.542^†^ |
| Inappropriate (≤24 points) | 196 (61.8) | 78 (63.9) | 118 (60.5) |  |
| Appropriate (≥25 points) | 121 (38.2) | 44 (36.1) | 77 (39.5) |  |
| How often do you sleep with mosquito nets?, *n* (%) |  |  |  | 0.214^‡^ |
| Always | 8 (2.5) | 1 (0.8) | 7 (3.6) |  |
| Frequently | 4 (1.3) | 0 (0) | 4 (2.1) |  |
| Sometimes | 18 (5.7) | 6 (4.9) | 12 (6.2) |  |
| Rarely | 23 (7.3) | 7 (5.7) | 16 (8.2) |  |
| Never | 264 (83.3) | 108 (88.5) | 156 (80) |  |
| How often do you sweep inside your home?, *n* (%) |  |  |  | 0.047^‡^ |
| Always | 235 (74.1) | 98 (80.3) | 137 (70.3) |  |
| Frequently | 35 (11) | 15 (12.3) | 20 (10.3) |  |
| Sometimes | 32 (10.1) | 7 (5.7) | 25 (12.8) |  |
| Rarely | 10 (3.2) | 2 (1.6) | 8 (4.1) |  |
| Never | 5 (1.6) | 0 (0) | 5 (2.6) |  |
| How often do you clean around your home?, *n* (%) |  |  |  | 0.822^†^ |
| Always | 74 (23.3) | 26 (21.3) | 48 (24.6) |  |
| Frequently | 107 (33.8) | 43 (35.2) | 64 (32.8) |  |
| Sometimes | 103 (32.5) | 39 (32) | 64 (32.8) |  |
| Rarely | 26 (8.2) | 10 (8.2) | 16 (8.2) |  |
| Never | 7 (2.2) | 4 (3.3) | 3 (1.5) |  |
| How often do you use insecticides inside your home?, *n* (%) |  |  |  | 0.401^†^ |
| Always | 46 (14.5) | 16 (13.1) | 30 (15.4) |  |
| Frequently | 35 (11) | 9 (7.4) | 26 (13.3) |  |
| Sometimes | 79 (24.9) | 31 (25.4) | 48 (24.6) |  |
| Rarely | 50 (15.8) | 19 (15.6) | 31 (15.9) |  |
| Never | 107 (33.8) | 47 (38.5) | 60 (30.8) |  |
| How often do you fumigate outside your home?, *n* (%) |  |  |  | 0.831^†^ |
| Always | 11 (3.5) | 5 (4.1) | 6 (3.1) |  |
| Frequently | 21 (6.6) | 9 (7.4) | 12 (6.2) |  |
| Sometimes | 58 (18.3) | 22 (18) | 36 (18.5) |  |
| Rarely | 78 (24.6) | 26 (21.3) | 52 (26.7) |  |
| Never | 149 (47) | 60 (49.2) | 89 (45.6) |  |
| When preparing natural fruit juices, do you ensure the fruit is clean?, *n* (%) |  |  |  | 0.662^‡^ |
| Always | 276 (87.1) | 109 (89.3) | 167 (85.6) |  |
| Frequently | 27 (8.5) | 7 (5.7) | 20 (10.3) |  |
| Sometimes | 8 (2.5) | 3 (2.5) | 5 (2.6) |  |
| Rarely | 4 (1.3) | 2 (1.6) | 2 (1) |  |
| Never | 2 (0.6) | 1 (0.8) | 1 (0.5) |  |
| When preparing natural fruit juices, do you ensure the blender is clean?, *n* (%) |  |  |  | 0.212^‡^ |
| Always | 276 (87.1) | 111 (91) | 165 (84.6) |  |
| Frequently | 29 (9.1) | 8 (6.6) | 21 (10.8) |  |
| Sometimes | 10 (3.2) | 2 (1.6) | 8 (4.1) |  |
| Rarely | 1 (0.3) | 0 (0) | 1 (0.5) |  |
| Never | 1 (0.3) | 1 (0.8) | 0 (0) |  |

^*^Mann-Whitney U test, ^†^chi-square test, ^‡^Fisher’s exact test. WRA: women of reproductive age, IQR: interquartile range
